# Efficacy of 1% Povidone-Iodine in the Treatment of Anterior Blepharitis—Randomized Single-Center Controlled Trial

**DOI:** 10.3390/jcm13237227

**Published:** 2024-11-28

**Authors:** Elishai Assayag, Adi Abulafia, David Teren, Evgeny Gelman, Hila Givoni, David Zadok

**Affiliations:** Department of Ophthalmology, Shaare Zedek Medical Center, Faculty of Medicine, Hebrew University of Jerusalem, Jerusalem 9103102, Israel

**Keywords:** blepharitis, povidone-iodine, dry eyes

## Abstract

**Background:** Anterior blepharitis (AB) is a chronic eyelid inflammation with no definitive cure. **Objectives:** To assess the safety and efficacy of a 1% povidone-iodine (PVI) ophthalmic solution lid scrub compared to formulated eyelid wipes in treating AB. **Design:** A prospective, randomized, controlled, observer-masked, paired-eye trial. **Methods:** Sixty-three AB patients were randomly assigned to a 30-day treatment in which one eye underwent a daily lid scrub with 1% PVI solution (1% PVI group), while the fellow eye was treated with formulated eyelid wipes (control group). Clinical outcomes, such as blepharitis signs, tear breakup time, and corneal staining, were evaluated at study enrollment and exit visits. Symptom assessments utilized the visual analog scale (VAS) per eye and the ocular surface disease index (OSDI) questionnaire. **Results**: Fifty-two patients (mean age 62.3 years, 53.8% females) completed the treatment, while seven patients were lost to follow-up, three were non-compliant, and one sustained an eye trauma. After 30 days, both the 1% PVI and control groups exhibited significant improvements in symptoms, blepharitis signs, and corneal staining (*p* < 0.05). The 1% PVI scrubs were equally effective as eyelid wipes in most outcomes (*p* = 0.480) and superior in alleviating eyelid erythema (*p* = 0.007). Only the 1% PVI group showed a positive correlation between OSDI and VAS score improvements (*r* (52) = 0.353, *p* = 0.01). No adverse events related to either treatment modality were reported. **Conclusions:** A 1% PVI solution is an effective, safe, and well-tolerated treatment option for AB and is superior to formulated eyelid wipes in several subjective and objective measures.

## 1. Introduction

Blepharitis is a chronic and frequently encountered inflammation of the eyelid margins, skin, and eyelashes. Common symptoms include ocular surface irritation, tearing, and redness. Eyelid erythema, crusting of lashes, dry eyes, eyelash distention or loss, meibomian gland clogging, and corneal damage are often observed on examination [1]. According to some reports, blepharitis is prevalent among over 35% of the population [2] and patients of all ages may be affected.

Blepharitis can be classified by the primary anatomic site of inflammation: anterior (skin and eyelashes) or posterior (meibomian gland dysfunction) [3]. Clinical presentations combining both forms are not uncommon. Anterior blepharitis (AB) is further categorized by its pathogenic etiology: bacterial (staphylococcal), parasitic (*Demodex*), or fungal (seborrheic) [4]. The overgrowth of these microorganisms on eyelid structures facilitates a local inflammatory response and tear-film derangements.

The mainstay of treating AB is preserving regular hygiene of the eyelids and lashes with formulated eyelid wipes, lid scrubbing solutions, baby shampoo, or tea tree oil-based cleansers [5]. Addition of a topical antibiotic, anti-parasitic, or steroidal agent is occasionally required [6]. Lotilaner ophthalmic solution 0.25% (XDEMVY, Tarsus Pharmaceuticals Inc., Irvine, CA, USA) was recently approved for treating *Demodex* blepharitis [6]. However, there is no definitive cure for AB, and some of the treatments mentioned above may have adverse effects, such as bacterial resistance, dermal or corneal toxicity, and raised intraocular pressure [7].

Povidone-iodine (PVI) is a commonly utilized water-soluble antiseptic agent with proven potency against a wide range of bacteria, viruses, and fungi [8]. The free iodine molecules penetrate pathogens and cause oxidative damage, which leads to apoptosis. In ophthalmology, PVI is regularly used to disinfect the ocular surface and periocular area before intravitreal injections, intraocular surgeries, and other procedures [9]. Furthermore, blepharitis is acknowledged as a risk factor for postoperative endophthalmitis, supporting most guidelines and surgeons to deem pre-operative antisepsis with PVI an essential preventative measure [10]. Certain practitioners employ low-concentration PVI formulations that generate lower corneal toxicity yet remain highly potent, owing to the abundance of free iodine molecules in the solution [9].

Although the pathophysiology of AB is at least partially infectious, only anecdotal studies have mentioned PVI as a treatment option [11,12]. In this study, we aimed to compare the safety and efficacy of eyelid scrubbing with 1% PVI ophthalmic solution to formulated eyelid wipes in treating AB.

## 2. Materials and Methods

### 2.1. Participants and Enrollment

This prospective, randomized, controlled, observer-masked, paired-eye clinical trial (ClinicalTrials.gov ID: NCT05160623) adhered to the tenets of the Declaration of Helsinki and was approved by the Institutional Review Board (IRB) of Shaare Zedek Medical Center, Jerusalem, Israel, on 8 December 2020 (Ethics Code: SZMC-20-0117). Subjects were referred to the study from outpatient and community clinics and through printed and social media advertisements. All patients provided written informed consent after being found eligible.

**Figure 1:**
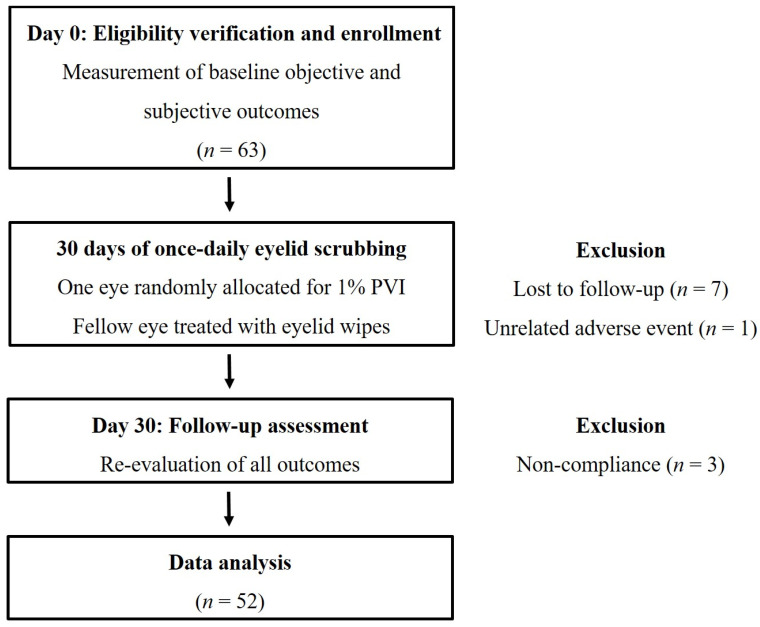
Trial flowchart.

The trial stages are summarized in Figure 1. To be included in the trial, participants had to be 18 years or older, with symptomatic anterior blepharitis evident on slit-lamp examination in the form of crusting of lashes, madarosis, telangiectatic erythema, or eyelid edema. Excluded were patients with another acute or chronic ocular surface disease, past eye surgery, regular use of contact lenses in the preceding 6 months, glaucoma treatment initiated or changed in the last month, known or suspected allergy to iodine, reluctance to avoid eyelash makeup for the duration of the study, pregnancy or lactation, and any medications (steroids, antibiotics, anti-inflammatories, isotretinoin) or conditions (i.e., rosacea, Sjögren syndrome) that may affect the ocular surface.

Eyelash crusting severity (graded between 0–4; see outcomes section below) was chosen as the outcome for sample size estimation. Fifty-one patients were required to have completed the trial for detecting a mean improvement of 0.5 points in crusting severity with a standard deviation (SD) of 0.8 [13]. The sample size calculation relied upon paired-eye comparisons with 95% power and a 2-sided significance level of 0.05 (5% probability of a type-I error).

### 2.2. Study Groups

The patients were asked to cease any current blepharitis and dry eye treatment, apart from artificial tears, for at least 2 weeks before enrollment. After enrollment and the first assessment of all outcomes, the patients received identical sets of materials and consumables and began the 30-day treatment consisting of one eye being randomly allocated to a once-daily eyelid scrubbing with 1% PVI ophthalmic solution (Concept Pharmacy, Kfar-Saba, Israel) applied with a makeup remover pad (1% PVI group). The fellow eye was scrubbed once daily with a formulated eyelid wipe (EYE-CARE forte, Dr. Fischer, Petah-Tikva, Israel) and served as the control group.

Before treatment initiation, best-corrected visual acuity, intraocular pressure, and dilated fundus examinations were performed to rule out underlying ocular pathologies. The patients received a sealed envelope that indicated which eye was allocated for treatment with 1% PVI and were instructed to treat the fellow eye with eyelid wipes. Each participant received detailed instructions on the scrubbing process. Patients were contacted two weeks into the treatment to verify their compliance and adherence to the instructions provided. The examiner was masked from treatment allocation. After 30 days of treatment, the subjects returned for a follow-up assessment, in which the same masked examiner (E.A.) re-evaluated all subjective and objective outcomes. Once all data were collected, each patient revealed which eye had been treated with 1% PVI.

### 2.3. Outcomes

The primary clinical outcome was the severity of blepharitis signs on a slit-lamp examination. Each sign (eyelid edema, erythema, and crusting of eyelashes) was graded separately in each eye as normal (0), mild (1), moderate (2), severe (3), or very severe (4), according to an illustrated scale used in previous studies [14,15]. Secondary clinical outcomes were tear breakup time (TBUT) and corneal staining, according to the National Eye Institute grading system (0–15 points) [16]. Corneal staining was also the primary safety parameter of this study.

Subjective data on blepharitis severity were collected from patients at study initiation and conclusion by completing the Ocular Surface Disease Index (OSDI) questionnaire (0–100 score) [17] and by self-grading overall ocular surface discomfort in each eye, employing a visual analog scale (VAS, 0–10 cm) [18]. The OSDI score estimates overall ocular surface symptoms without distinguishing between eyes. Therefore, it was not used to compare between study groups directly but in correlation to VAS score changes in each eye.

### 2.4. Statistical Analysis

Statistical analyses were performed using Microsoft Excel 2019 (Microsoft Corporation, Redmond, WA, USA). Quantitative variables were summarized as means and standard deviations. All datasets were first tested for normality of distribution using the Kolmogorov–Smirnov test. Outcome differences between day 1 and day 30 and between treatment groups were estimated with 2-tailed paired Student’s *t*-tests. Correlations were computed with a Pearson correlation coefficient. A *p*-value < 0.05 was considered significant.

## 3. Results

### 3.1. Study Population

Sixty-three patients who met the inclusion criteria were enrolled in the study, and 52 (82.5%) completed the required 30-day treatment, thus exceeding the minimum sample size. Reasons for non-completion included seven patients lost to follow-up, three non-compliant patients, and one patient who sustained an eye trauma during the treatment.

The demographic and baseline characteristics of participants who were eventually included in the analysis are depicted in Table 1. The average age of the patients was 62.3 ± 17.2 years, with females comprising 53.8% (*n* = 28) of the cohort.

### 3.2. Objective and Safety Outcomes

**Figure 2:**
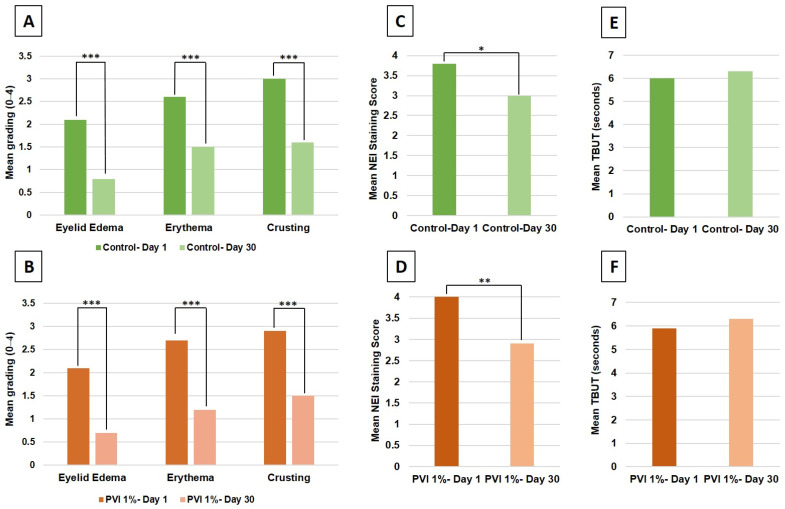
Objective outcomes in the 1% PVI and control groups after 30 days. (**A**,**B**) The severity of blepharitis signs (0–4 Efron scale) on slit-lamp exam. (**C**,**D**) Corneal staining according to the NEI grading system (0–15 points). (**E**,**F**) TBUT (seconds). Means of baseline vs. day 30 findings were compared using a two-tailed paired Student’s *t*-test. Error bars represent the standard deviation. * *p* < 0.05, ** *p* < 0.01, *** *p* < 0.001. NEI = National Eye Institute; TBUT = tear breakup time.

Following 30 days of treatment, both groups exhibited significant improvements across all mean grades of blepharitis signs (Figure 2A,B). Specifically, the eyelid edema grades improved (1% PVI, from 2.1 to 0.7, *p* < 0.001; control, from 2.1 to 0.8, *p* < 0.001), as did those for erythema (1% PVI, from 2.7 to 1.2, *p* < 0.001; control, from 2.6 to 1.5, *p* < 0.001), and for crusting (1% PVI, from 2.9 to 1.5, *p* < 0.001; control, from 3.0 to 1.6, *p* < 0.001). Additionally, a more pronounced reduction in mean corneal staining was observed in the 1% PVI group (Figure 2C,D: 1% PVI, from 4.1 to 2.9, *p* = 0.004; control, from 3.8 to 3.0, *p* = 0.041). There was no significant change in mean TBUT in either of the study groups (Figure 2E,F: 1% PVI, from 5.9 to 6.3, *p* = 0.380; control, from 6.0 to 6.3, *p* = 0.460).

**Figure 3:**
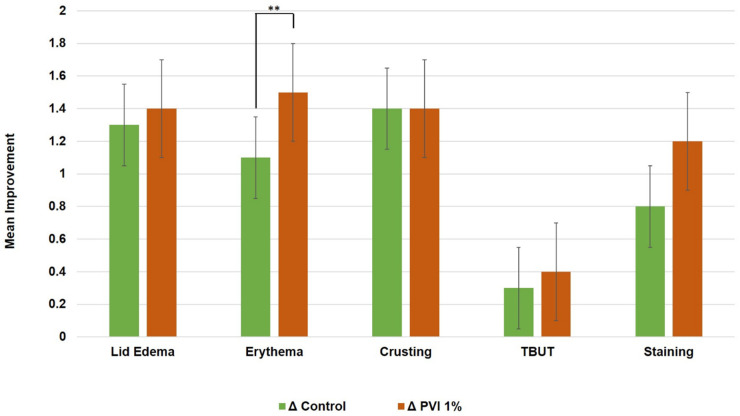
Paired-eye comparisons of improvements in objective outcomes. Means of improvement values in the 1% PVI group vs. the control group were compared using a two-tailed paired Student’s *t*-test. Error bars represent the standard deviation. ** *p* < 0.01. TBUT = tear breakup time.

A paired-eye comparison revealed that the 1% PVI and control groups showed comparable mean improvements in most outcomes (Figure 3). These outcomes included eyelid edema (1.4 vs. 1.3, *p* = 0.480), crusting (1.4 vs. 1.4, *p* = 0.636), corneal staining (1.2 vs. 0.8, *p* = 0.263), and TBUT (0.4 vs. 0.3, *p* = 0.954). Notably, 1% PVI treatment resulted in a significantly enhanced mean improvement in eyelid erythema compared to the formulated eyelid wipes (1.5 vs. 1.1, respectively, *p* = 0.007). Finally, no adverse events associated with either treatment were reported throughout the trial.

### 3.3. Subjective Outcomes

**Figure 4:**
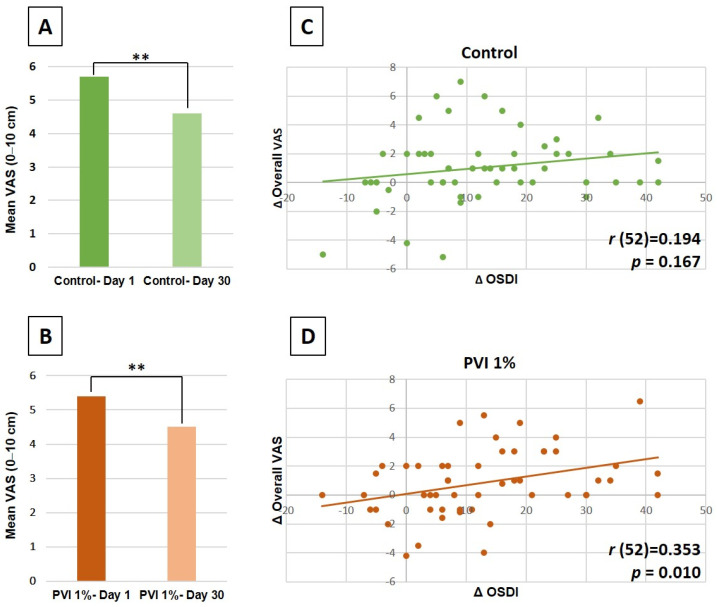
Subjective outcomes and correlations to treatments. (**A**,**B**) Mean VAS scores of overall ocular surface discomfort at baseline vs. day 30 compared by a 2-tailed paired Student’s *t*-test. Error bars represent the standard deviation. ** *p* < 0.01. (**C**,**D**) Correlations between the change in OSDI score and the improvement in VAS scores in each group. Correlations were computed with a Pearson correlation coefficient. VAS = visual analog scale; OSDI = ocular surface disease index.

On day 30, there was a significant reduction in the mean OSDI score compared to the baseline score (12.0 vs. 25.0, respectively, *p* < 0.001). Both treatment groups exhibited lower mean VAS scores for overall ocular surface discomfort (Figure 4A,B: 1% PVI, from 5.4 to 4.5, *p* = 0.009; control, from 5.7 to 4.6, *p* = 0.003). The mean reduction in VAS score was not significantly different between the 1% PVI and control groups (0.9 vs. 1.1, respectively, *p* = 0.520). However, the changes in the OSDI score were positively correlated with the improvements in VAS scores only in the 1% PVI group (Figure 4C,D, *r* (52) = 0.353, *p* = 0.01).

## 4. Discussion

We evaluated the safety and efficacy of a daily eyelid cleanse with a 1% PVI ophthalmic solution compared to commercial eyelid wipes for treating AB in a 30-day randomized controlled trial. A comparative analysis of objective outcomes between study enrollment and study closure demonstrated the efficacy of 1% PVI, particularly by a significant reduction in corneal staining. A paired-eye analysis revealed comparable effectiveness between the two treatments, but 1% PVI exhibited superiority in mitigating eyelid erythema. Lastly, an analysis of the subjective outcomes suggested that the marked alleviation in ocular surface discomfort was predominantly attributed to the eye treated with the 1% PVI solution.

Each subtype of AB is characterized by pathophysiological features linked to its respective causative microorganism. The inflammation in staphylococcal AB is believed to arise from irritation of the eyelid tissues by toxins produced by coagulase-negative staphylococcus and *S. aureus* [1]. An increased cell-mediated immune response to *S. aureus* has been proposed as another mechanism contributing to AB [19]. In *Demodex* blepharitis, the sebum-consuming mites induce inflammation by mechanically and biochemically compromising the eyelids [20] and indirectly acting as vectors for other pathogens that promote AB [21]. Seborrheic AB, often associated with seborrheic dermatitis, has been attributed to *Malassezia*, a genus of skin-inhibiting fungi [22].

The effectiveness of 1% PVI in reducing the severity of eyelid edema, erythema, and crusting, as demonstrated in this trial, may be ascribed to the antiseptic properties of PVI against the culpable pathogens, with PVI having been proven potent in eliminating *S. aureus* [23] and *S. epidermidis* [24], even in diluted preparations [25]. Various species of fungi are also vulnerable to different PVI concentrations [26,27]. Although specific data on *Malassezia* are scarce, we consider it reasonable to assume that, similar to other yeasts, it may be vulnerable to PVI.

The rationale for PVI as a treatment option for *Demodex* blepharitis is more complex. Only one study has documented that *Demodex* mites exhibited resistance to a 10% PVI solution in an in vitro environment [28]. However, to our knowledge, this resistance has neither been demonstrated in vivo nor explored within the framework of controlled clinical trials. Furthermore, Pelletier et al. observed that a 0.25% topical PVI formulation conferred substantial therapeutic benefits in persistent *Demodex* blepharitis following the failure of topical antibiotics, steroids, and tea tree oil-based treatments [12]. We suggest that despite the potential resistance of *Demodex* mites to PVI, some of their activity as indirect facilitators of bacterial AB is plausibly counteracted by the antiseptic effects of the solution.

It has been postulated that punctate epithelial corneal erosions in the eyes of patients with blepharitis result from tear-film alterations caused by inflammatory mediators, namely, exotoxins produced by *S. aureus*. These virulent factors are secreted directly by the bacteria and notably accumulate in eyelid margin bacterial biofilms, a distinctive feature of AB pathogenesis [29]. PVI has demonstrated efficacy in eliminating biofilms of *S. aureus* and *S. epidermidis* formed in other tissues [24]. Therefore, although no bacterial cultures were collected in our study, we hypothesize that the comparatively marked reduction of corneal staining observed in the 1% PVI group resulted from decreased bacterial colonization and potential eradication of biofilms along the eyelid margin.

Erythema of the eyelids is a frequently observed and particularly distressing symptom in patients diagnosed with blepharitis [2,30,31]. Erythema likely stems from an interplay between immune-mediated reactions and direct mechanical irritation, contributing to the formation of telangiectatic and engorged blood vessels. While PVI is well known for its antiseptic properties, several studies have suggested that it might also confer anti-inflammatory benefits by acting as a free radical scavenger, an oxidative stress stabilizer, and an attenuator of cytokine activity [32,33]. The small yet significant superiority of 1% PVI compared to commercial eyelid wipes in reducing eyelid erythema, as we observed, could be attributed to its specific capabilities.

TBUT is an important clinical sign in dry eye assessment and usually reflects tear-film instability due to insufficient quality or quantity of the lipid layer [34]. Since patients were asked to cease any other treatment for dry eyes during the study, we assume that the lack of improvement in TBUT in both groups indicates that compared treatments did not address possible meibomian gland dysfunction that patients may have had in addition to AB.

A recent study found that repeated ocular surface pre-operative irrigation with PVI 1% was superior to a single irrigation with PVI 5% in reducing corneal staining and conjunctival bacterial colony counts [35]. This difference was attributed to a greater bactericidal potency of low-concentration PVI formulas, which contain more free iodine molecules but require several applications to reach a maximal effect, as was seen in other in-vitro and in vivo studies [9]. It is possible that higher frequency regimens of treating AB with PVI 1%, compared to the once-daily scrubbing performed in our study, would be even more effective than the results we presented.

Several studies have indicated a 30–45% non-compliance rate among blepharitis patients in adhering to daily eyelid hygiene protocols, even within a controlled clinical trial environment [36,37]. Discontinuation or irregular usage of lid hygiene products may be attributed to various factors, including forgetfulness and perceived insufficiency of subjective improvement [36], as well as to adverse effects, such as skin dryness, ocular irritation, contact dermatitis, and the exacerbation of blepharitis symptoms [38]. In our study, the favorable correlation between the reduction of overall ocular surface discomfort and the reported subjective improvement in the 1% PVI group, coupled with the absence of PVI-related adverse events, bodes well for future patient compliance and satisfaction.

Our research revealed that the control treatment, consisting of daily scrubbing with commercial eyelid wipes, significantly improved most subjective and objective outcomes. The effectiveness of the wipes in treating AB may be attributed to specific ingredients, such as chamomile and glycerol, which are notably abundant in the formula, according to the manufacturer. Chamomile is recognized for its well-established curative properties, including anti-inflammatory and antimicrobial activity [39]. Similarly, topical glycerol has been documented for its beneficial role in managing various skin conditions [40]. Nevertheless, it is essential to consider potential adverse effects and costs associated with the regular use of eyelid wipes, as reported in some studies [38]. Lastly, the costs and limited availability of such products in developing regions highlight the necessity of alternative treatments.

Our study bears several limitations. Firstly, we neither collected bacterial cultures nor documented the presence of *Demodex*, precluding a sub-analysis of different AB subtypes. Stratifying the patients according to these factors in future research might clarify the mechanisms involved in PVI’s efficacy in each subtype. Secondly, additional safety and efficacy outcome measures, such as conjunctival staining score and other questionnaires, may have added value to the study. Thirdly, while patients employed identical ready-to-use formulated wipes, the 1% PVI solution required daily application onto a pad. However, given the paired-eye design of the trial, we believe that such disparities, interocular or intersubject, should not detrimentally influence the quality of our analysis. Finally, the paired-eye design introduces inherent challenges, such as a lack of patient masking and the potential for treatment confusion. We sought to mitigate the latter by reminding the patients to be vigilant throughout treatment.

In conclusion, our results indicate that once-daily eyelid scrubbing with a 1% PVI ophthalmic solution is an effective, safe, and well-tolerated treatment option for AB. Notably, 1% PVI demonstrated superiority over conventional eyelid hygiene with dedicated formulated wipes in several objective and subjective outcome measures. Further research is warranted to elucidate the impact of PVI on distinct AB subtypes, explore the efficacy of varied treatment frequencies, and assess its performance over longer follow-up durations.

## Figures and Tables

**Table 1:** Demographic and baseline characteristics of participants who completed the trial.

Characteristic		1% PVI Group (Mean ± SD)	Control Group (Mean ± SD)	*p*
Age, years, mean ± SD	62.3 ± 17.2			
Gender Male, n (%) Female, n (%)	24 (46.2) 28 (53.8)			
Subjective complaints OSDI score, 0–100 (mean ± SD) VAS score, 0–10 cm	25 ± 15.8	5.4 ± 0.8	5.7 ± 0.4	0.287
Blepharitis signs, Efron scale, 0–4 Eyelid edema Eyelid erythema Eyelid crusting		2.1 ± 0.7 2.7 ± 0.8 2.9 ± 0.7	2.1 ± 0.8 2.6 ± 0.7 3.0 ± 1.0	0.322 0.484 0.659
Dry eye measurements TBUT, seconds Corneal staining, NEI score, 0–15		5.9 ± 1.3 4.1 ± 1.2	6.0 ± 1.2 3.8 ± 1.3	0.706 0.381

PVI = povidone-iodine; SD = standard deviation; OSDI = ocular surface disease index; VAS = visual analog scale; TBUT = tear breakup time; NEI = National Eye Institute. Study groups were compared using a two-tailed paired Student’s *t*-test.

## Data Availability

The datasets generated and analyzed during the current study are available from the corresponding author upon reasonable request.
